# Case Report: Multiorgan arterial and venous thrombosis after ischemic stroke: endovascular management and subsequent transcatheter patent foramen ovale closure

**DOI:** 10.3389/fcvm.2026.1993549

**Published:** 2026-10-28

**Authors:** Eustaquio M. Onorato, Giovanni Monizzi, Angelo Mastrangelo, Vincenzo Mallia, Francesca Giacomazzi, Gian Paolo Anzola, Antonio L. Bartorelli

**Affiliations:** 1 University Cardiology Department, IRCCS Ospedale Galeazzi-Sant'Ambrogio, Milan, Italy; 2 Consultant Neurologist, Villa Barbarano Clinic, Salò, Italy; 3 Department of Biomedical and Clinical Sciences, University of Milan, Milan, Italy

**Keywords:** floating aortic thrombus, paradoxical embolism, patent foramen ovale, pulmonary embolism, superior mesenteric artery thrombosis

## Abstract

**Background:**

Concurrent cerebral, visceral, renal, aortic, and pulmonary thrombosis is rare and may reflect multiple mechanisms. Identifying the embolic source and balancing antithrombotic therapy against intracranial bleeding are major clinical challenges.

**Case summary:**

A 63-year-old woman presented on 3 February 2025 with right middle cerebral artery (MCA) M1 occlusion and underwent intravenous thrombolysis followed by mechanical thrombectomy, achieving Thrombolysis in Cerebral Infarction (TICI) grade 2b reperfusion. The procedure was complicated by self-limited M1 wall injury and right Sylvian subarachnoid hemorrhage, with subsequent right hemispheric infarction and intraparenchymal blood products. During rehabilitation, emergency imaging on 5 March 2025 demonstrated a saddle pulmonary embolism (PE) with bilateral extension, a floating abdominal aortic thrombus, occlusive superior mesenteric artery (SMA) thrombosis with preserved bowel perfusion, hepatic and pancreaticoduodenal arterial thrombosis, renal embolic lesions, and new left hemispheric and cerebellar ischemic lesions. Aortic mechanical thrombectomy was performed with a Penumbra Indigo system and a Lightning 7-Fr catheter; persistent proximal SMA thrombus was treated with two balloon-expandable Omnilink stents (8 × 59 mm and 8 × 39 mm). Intravenous unfractionated heparin was transitioned to warfarin on 12 March 2025. In March 2026, contrast transcranial Doppler documented a large right-to-left shunt after the Valsalva maneuver, and two-dimensional (2D) transthoracic and transesophageal echocardiography (TTE/TEE) with color Doppler confirmed a tunnel-like patent foramen ovale (PFO) with a septum primum aneurysm. On 24 March 2026, rotational intracardiac echocardiography (rICE)-guided closure was performed under local anesthesia using a II-Hubless 22/28 mm Memopart PFO Occluder (LEPU Medical).

**Discussion:**

The records support concurrent arterial and venous thrombosis, not a single embolic cascade. The aortic thrombus plausibly caused visceral and renal embolization but not PE; the later PFO represented a potential, unproven route for paradoxical embolism. Despite substantial clot burden and RV dysfunction, incomplete data precluded reliable retrospective ESC risk classification. PFO closure was an individualized adjunct to anticoagulation given the patient's age, alternative embolic sources, and incomplete prolonged rhythm monitoring.

## Highlights

- Evaluate concurrent pulmonary and systemic arterial embolism for distinct venous, cardiac, and aortic sources, including a right-to-left shunt.
- Balance reperfusion and anticoagulation against intracranial bleeding risk through multidisciplinary, individualized decision-making.
- Consider PFO closure an individualized adjunct to anticoagulation only after assessing competing embolic mechanisms, age, and rhythm-monitoring findings.

## Introduction

Concurrent pulmonary embolism and systemic arterial ischemia may reflect independent venous and arterial thromboses, aortic embolization, or paradoxical embolism through a right-to-left shunt (RLS). Floating aortic thrombus is an uncommon source of peripheral embolization, whereas a patent foramen ovale (PFO) is a potential conduit rather than proof of paradoxical embolism. We report cerebral, aortic, visceral, renal, and pulmonary thrombosis requiring staged multidisciplinary management, highlighting diagnostic uncertainty, competing thrombotic and bleeding risks, and the need for cautious causal interpretation.

## Case presentation

A 63-year-old woman with remote tobacco use, dyslipidemia, borderline glucose values, celiac disease, prior postoperative deep vein thrombosis, two spontaneous abortions, and recurrent intestinal adenomas presented on 3 February 2025 with acute left hemiparesis. CTA showed right MCA M1 occlusion. Intravenous thrombolysis was followed by mechanical thrombectomy; after three combined stent-retriever and aspiration passes, TICI 2b reperfusion was achieved in the frontoparietal division, while the anterior frontal division remained occluded (5). Self-limited M1 wall injury/dissection and right Sylvian subarachnoid hemorrhage complicated the procedure, followed by right fronto-insular and fronto-temporo-parietal infarction with intraparenchymal blood products and transient mass effect (Figure 1). During rehabilitation, syncope during mobilization with diarrhea was followed by gastrointestinal symptoms, fever, inflammatory-marker elevation, and markedly elevated D-dimer. On 4 March, ultrasonography suggested floating abdominal aortic thrombi, prompting urgent CTA.

**Figure 1:**
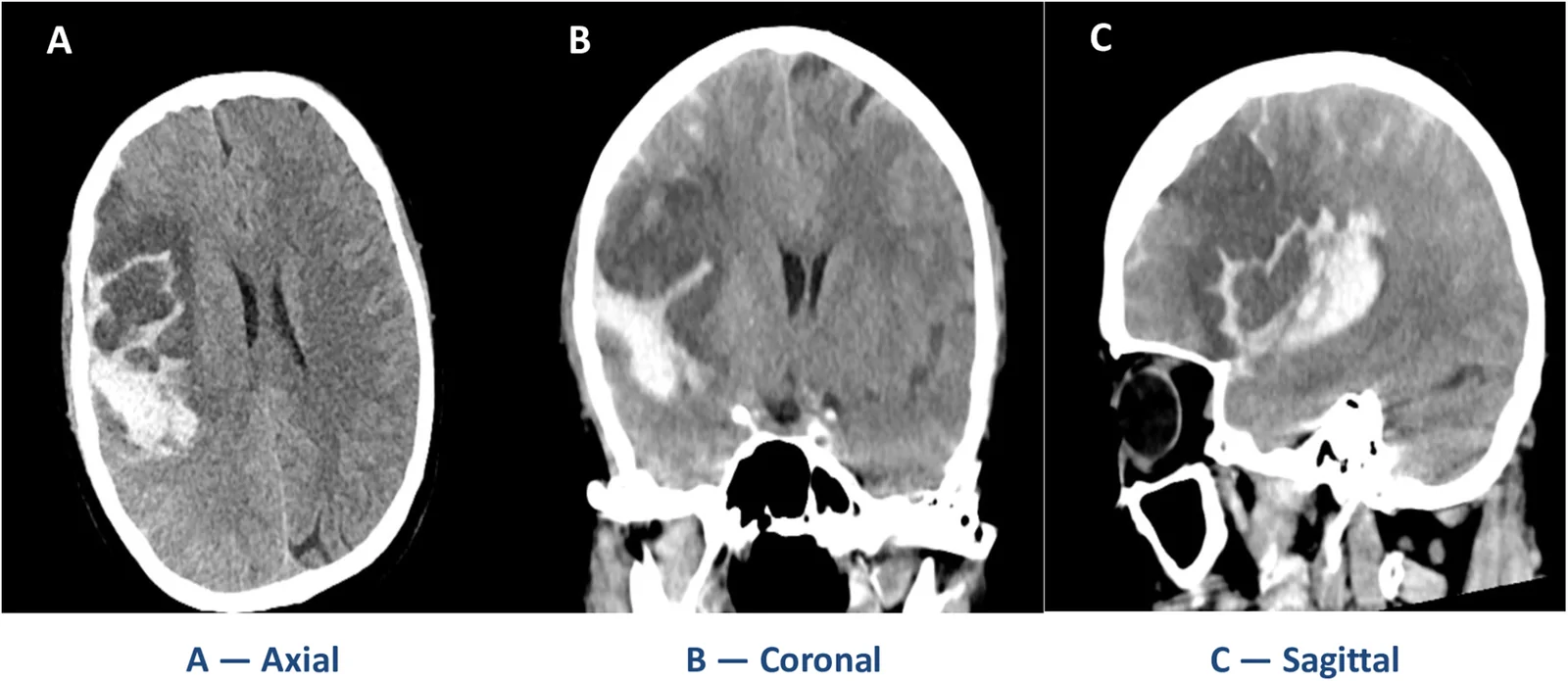
Brain computed tomography after reperfusion treatment. Multiplanar non-contrast CT images in axial **(A)**, coronal **(B)**, and sagittal **(C)** planes demonstrate right fronto-temporo-parietal ischemic injury with hemispheric swelling and mass effect. Hyperdense blood products are present in the right Sylvian subarachnoid space and within the brain parenchyma.

CTA performed on 5 March 2025 confirmed a saddle PE with bilateral lobar and segmental extension, a floating abdominal aortic thrombus, occlusive SMA thrombosis with preserved bowel perfusion, subocclusive thrombus in the common hepatic artery and at the origin of the pancreaticoduodenal arteries, renal arterial thrombi and renal ischemic lesions, without computed tomography (CT) evidence of bowel ischemia (Figures 2–4). The preceding ultrasound had described two rounded hyperechoic mobile intraluminal aortic formations measuring approximately 12 mm and 5 mm, interpreted as floating thrombi.

**Figure 2:**
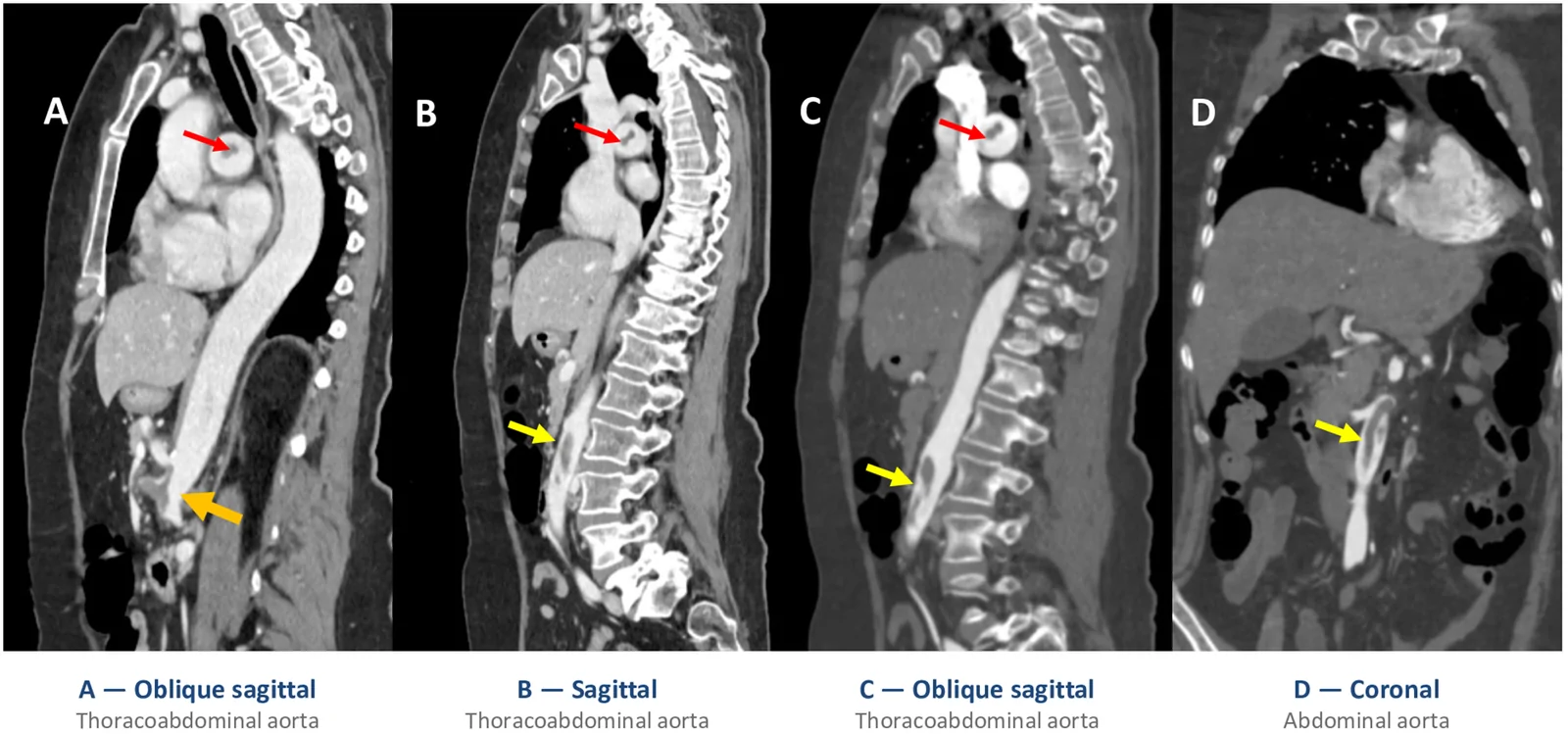
Thoracoabdominal computed tomography angiography showing aortic and visceral arterial thrombosis. **(A)** Oblique sagittal reconstruction showing an occlusive thrombus at the origin of the superior mesenteric artery (orange arrow). **(B)** Sagittal reconstruction showing a mobile thrombus in the abdominal aorta (yellow arrow). **(C)** Oblique sagittal reconstruction showing the abdominal aortic thrombus (yellow arrow) and pulmonary arterial filling defects (red arrows). **(D)** Coronal reconstruction showing the abdominal aortic thrombus (yellow arrow) and pulmonary arterial filling defects (red arrows).

**Figure 3:**
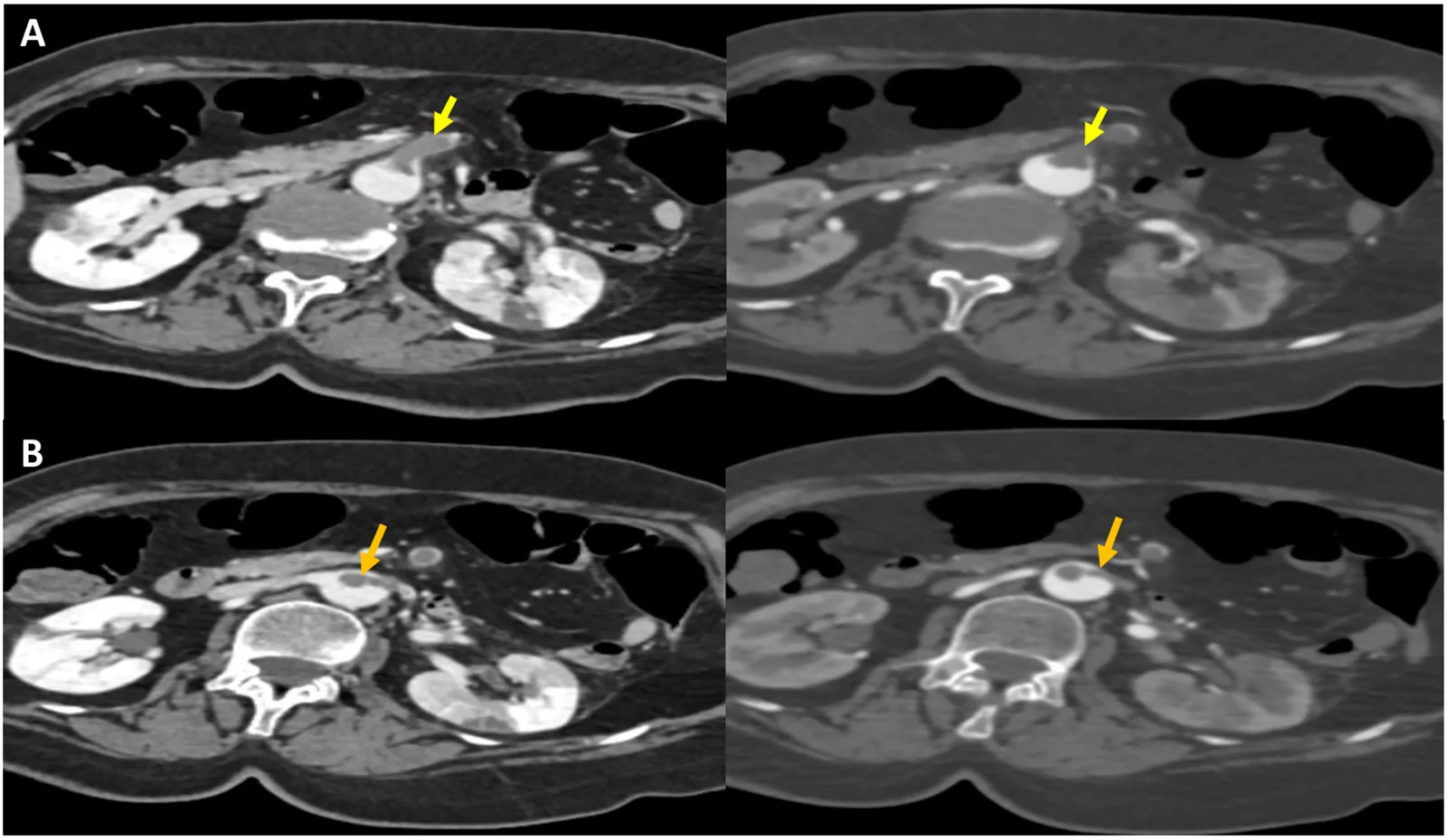
Contrast-enhanced computed tomography angiography of the abdomen. Axial arterial-phase images show **(A)** an occlusive thrombus at the origin of the superior mesenteric artery (yellow arrows) and **(B)** a thrombus adjacent to the origin of the right renal artery (orange arrow), with multiple peripheral wedge-shaped hypoattenuating areas in the right kidney consistent with renal infarction.

**Figure 4:**
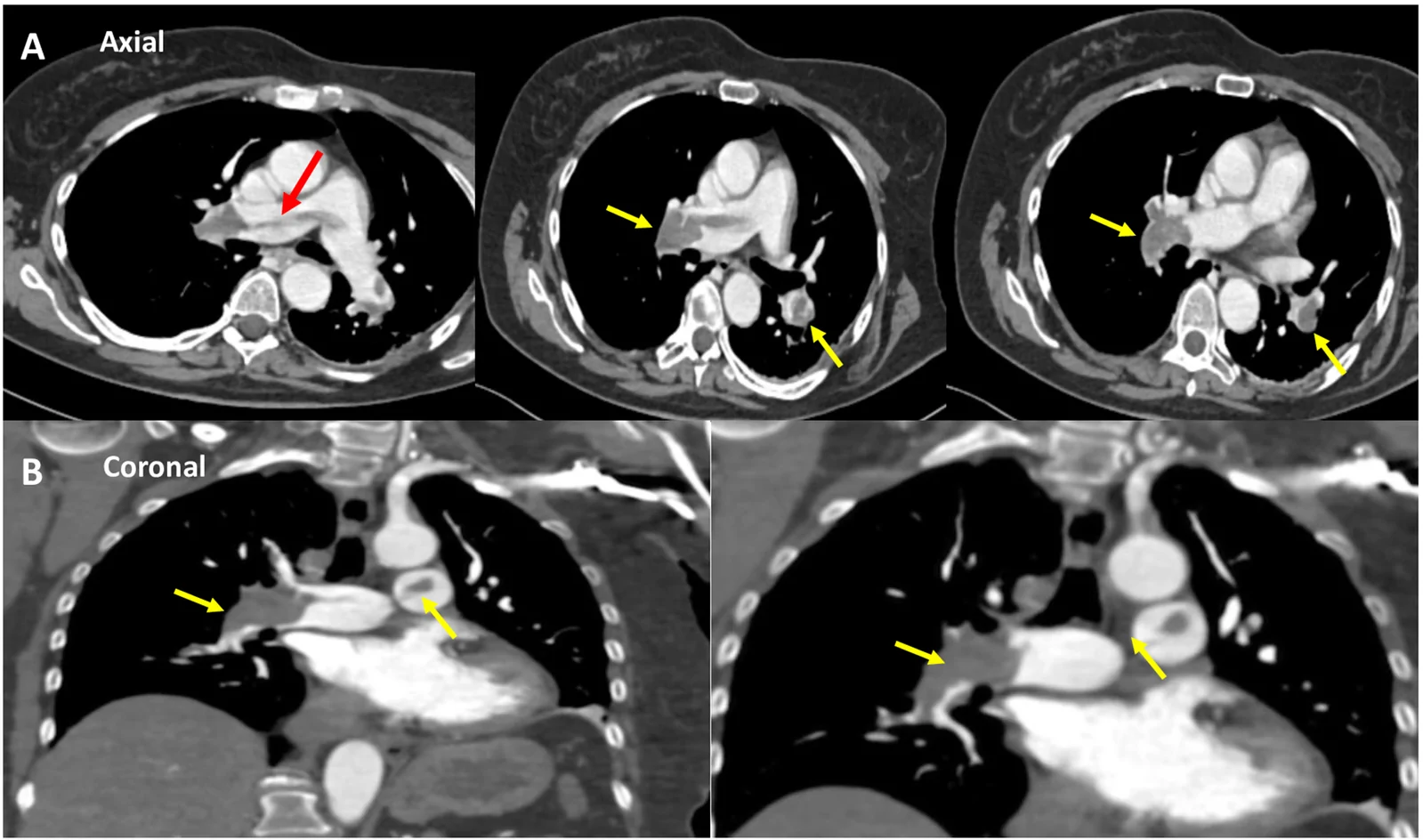
Computed tomography pulmonary angiography demonstrating saddle pulmonary embolism. **(A)** Axial images show a saddle filling defect at the main pulmonary arterial bifurcation, with extension into both main pulmonary arteries and the lobar and segmental branches bilaterally. **(B)** Coronal reconstructions show thrombus within the central pulmonary arteries bilaterally, extending into the lobar and segmental branches. The red arrow indicates the saddle component; yellow arrows indicate additional central and branch pulmonary arterial filling defects.

2D TTE showed RV dysfunction, interventricular septal flattening, a TAPSE of 14 mm, and an estimated PASP of approximately 75 mmHg. At vascular consultation, the patient was awake, oriented, and hemodynamically stable. Venous blood gas analysis on 5 March 2025 showed a lactate concentration of 0.8 mmol/L.

Intravenous unfractionated heparin was started. Emergency endovascular treatment was performed on 5 March 2025 with anesthesiology support and bilateral ultrasound-guided common femoral arterial access. Diagnostic aortography confirmed a large floating infrarenal abdominal aortic thrombus extending cranially toward the SMA ostium and proximal segment (Supplementary Figure S1). Mechanical thrombectomy of the aortic component was performed with a Penumbra Indigo system and a Lightning 7-Fr catheter; attempts to mobilize or remove the mesenteric component remained incomplete. Because abundant non-recent thromboembolic material persisted in the proximal SMA, two balloon-expandable Omnilink stents (8 × 59 mm and 8 × 39 mm) were implanted, restoring SMA patency, a regular vessel contour, and distal perfusion.

Neurologic status remained stable in the ICU, and the patient was extubated the next day without respiratory distress. Heparin was continued until 12 March 2025, then replaced by warfarin. Ultrasonography on 14 March showed a patent SMA stent and no residual aortic thrombus. One-month brain CT demonstrated evolution of the right hemispheric infarction, persistent intraparenchymal blood products, and near-resolution of mass effect (Supplementary Figure S2). Acute kidney injury peaked at a serum creatinine level greater than 6 mg/dL, improving to 3.55 mg/dL at ICU transfer on 14 March and 1.06 mg/dL by 24 March. Acute cholecystitis was treated conservatively with ceftriaxone; asymptomatic Pseudomonas aeruginosa bacteriuria required only catheter management. She returned to rehabilitation on 25 March 2025.

Etiologic testing was negative for Factor V Leiden, prothrombin G20210A, anticardiolipin IgG/IgM, anti-β2-glycoprotein I IgG/IgM, ANA, ANCA, and a monoclonal serum component. Venous duplex showed no acute deep vein thrombosis but chronic segmental thrombosis of the left small saphenous vein. Inpatient ECG monitoring showed sinus rhythm; a monitor-detected atrial fibrillation episode was not confirmed by cardiology review or concomitant ECG. A planned 24 h Holter report was unavailable.

No dedicated RLS assessment was documented until March 2026, when c-TCD showed a large “curtain” shunt after Valsalva. Subsequent 2D TTE/TEE with color Doppler identified a tunnel-like PFO measuring 9 × 5 mm with a septum primum aneurysm. From a diagnostic perspective, clinical consensus dictates that c-TCD serves as the premier modality for RLS detection and grading due to its exceptional sensitivity to microembolic signals in the cerebral circulation; conversely, echocardiography excels in providing the critical anatomical localization and structural morphology required to differentiate intracardiac from intrapulmonary shunts.

The heart–brain team recommended PFO closure as an individualized adjunct to anticoagulation, considering the arterial and venous events, documented RLS, age, alternative embolic sources, incomplete prolonged rhythm monitoring, and uncertain incremental benefit during anticoagulation. Warfarin was withheld for 3 days before admission on 23 March 2026. On 24 March, closure was performed under local anesthesia through bilateral femoral venous access with rICE guidance. rICE confirmed the tunnel-like PFO and septum primum aneurysm. A II-Hubless 22/28 mm Memopart PFO Occluder (LEPU Medical) was implanted (Supplementary Figure S3). Femoral-vein agitated-saline injection showed no residual shunt, and no complication occurred.

Post-procedural TTE confirmed correct device position without pericardial effusion. Warfarin was restarted on 25 March 2026 with enoxaparin 6,000 IU twice daily until INR exceeded 2; subsequent dosing was managed by the anticoagulation clinic. At discharge, serum creatinine was 1.00 mg/dL and platelet count 253 × 10^9^/L. TTE and cardiology follow-up were planned at 6 and 12 months and annually. The March 2025 discharge record specified an INR target of 2–3, but anticoagulation duration, rationale for continued warfarin, antiplatelet therapy after SMA stenting or PFO closure, and later shunt and pulmonary-pressure measurements were undocumented.

## Discussion

This case documents thrombosis in multiple arterial and venous beds without establishing a single embolic cascade. The abdominal aortic thrombus plausibly caused the mesenteric, hepatic, renal, and possibly some cerebral events but not the PE. The new left hemispheric and cerebellar lesions occurred during the systemic thrombotic presentation without identifying a unique source. The later PFO diagnosis indicated a potential conduit for paradoxical embolism during right-sided pressure overload but did not prove transseptal thrombus passage. Independent arterial and venous thromboses from an acquired (6–9), transient, or incompletely characterized prothrombotic state remain plausible.

Floating aortic thrombus is rare in the absence of aneurysm, severe atherosclerosis, or trauma (1), and is associated with substantial embolic risk (2). In this patient, percutaneous mechanical thrombectomy offered rapid source control while avoiding open surgery, and SMA stenting restored mesenteric patency after unsuccessful aspiration (3, 4). The absence of bowel ischemic complications supports the relevance of timely endovascular revascularization in selected patients.

The saddle PE extended bilaterally into lobar and segmental branches, with RV dysfunction, septal flattening, TAPSE 14 mm, and estimated PASP approximately 75 mmHg. Nevertheless, clot burden does not define hemodynamic risk, which requires integration of hemodynamic status, clinical risk scores, RV dysfunction, and cardiac biomarkers (10). The patient was stable at vascular consultation; venous lactate was 0.8 mmol/L on 5 March, and subsequent arterial values were mainly 0.5–1.0 mmol/L, with one value of 2.1 mmol/L on 13 March. Troponin, natriuretic peptides, RV/LV ratio, and complete PESI/sPESI variables were unavailable, precluding reliable retrospective ESC classification. Earlier patient data reported reduced-dose tenecteplase, but administration, dose, timing, indication, neurological assessment, pre-treatment imaging, deterioration, participating specialties, and rationale were unverified. The manuscript therefore reports only documented heparin followed by warfarin and does not present systemic reperfusion as confirmed therapy. Observational evidence regarding reperfusion therapy in patients with acute PE and right-heart thrombi further underscores the need for individualized treatment decisions (11).

The causal pathway remains uncertain. Potential sources include the aortic thrombus, paradoxical embolism through the PFO, occult atrial fibrillation, and an unrecognized prothrombotic condition. Atrial fibrillation was not documented in hospital but could not be excluded without prolonged monitoring. No dedicated RLS or PFO evaluation was documented before March 2026; this limitation does not establish that earlier detection or closure would have prevented the events. Evidence for PFO closure is strongest in selected patients aged 18–60 years with adjudicated PFO-associated stroke (12, 13). In this older patient with vascular risk factors and an ongoing indication for anticoagulation, closure was an individualized heart–brain-team decision rather than guideline-mandated therapy.

Negative Factor V Leiden, prothrombin G20210A, antiphospholipid antibody, ANA, ANCA, and serum electrophoresis results narrowed but did not exclude acquired or transient thrombophilia; lupus anticoagulant, protein C, protein S, antithrombin, malignancy evaluation, and assay timing relative to anticoagulation were undocumented. Platelets fell from 322 × 10^9^/L on 14 February to 110 × 10^9^/L on 3 March, before therapeutic heparin began, and then recovered. No 4Ts assessment, platelet factor 4 (PF4)/heparin antibody test, or functional heparin-induced thrombocytopenia (HIT) assay was available. Anticoagulation remained central to secondary prevention; PFO closure addressed only a potential paradoxical embolic route.

### Strengths and limitations

Strengths include a source-verified chronology, multimodality imaging, detailed endovascular records, and multidisciplinary management. Limitations include absent thrombus histopathology; incomplete characterization of the aortic attachment site and wall; no documented dedicated RLS assessment before March 2026; incomplete thrombophilia, HIT, malignancy, and rhythm evaluation; absence of verified pre-closure RV and pulmonary-pressure recovery, formal assessment for chronic thromboembolic pulmonary hypertension, and later quantitative shunt and pulmonary-pressure data; insufficient data for retrospective ESC PE risk classification; and inability to verify previously reported tenecteplase treatment for PE. The independent effects of anticoagulation and PFO closure cannot be determined.

## Conclusions

This case highlights the complexity of multiterritorial arterial and venous thrombosis after ischemic stroke. Aortic thrombectomy, SMA stenting, and anticoagulation were followed by mesenteric and renal recovery, although their individual effects cannot be isolated. The aortic thrombus was a plausible arterial source but did not explain the PE; the later PFO diagnosis indicated a potential, unproven paradoxical route. In this older anticoagulated patient with competing mechanisms, PFO closure was an individualized adjunct rather than proof of causation.

## Patient perspective

The patient described the prolonged sequence of neurological, vascular, and cardiac procedures as physically and emotionally demanding. She considered the recovery of organ function and continued rehabilitation to be the most important outcomes and understood that PFO closure addressed only one potential embolic pathway, while anticoagulation remained an essential component of her management. This statement was reviewed and approved by the patient.

## Data Availability

The raw data supporting the conclusions of this article will be made available by the authors, without undue reservation.
